# Clinical Evaluation of the Immunochromatographic System Using Silver Amplification for the Rapid Detection of *Mycoplasma pneumoniae*

**DOI:** 10.1038/s41598-018-19734-y

**Published:** 2018-01-23

**Authors:** Ho Namkoong, Masahiko Yamazaki, Masami Ishizaki, Ikumi Endo, Noriaki Harada, Megumi Aramaki, Yuko Tanaka, Sachiko Kaburagi, Masataka Ichikawa, Takanori Ohata, Shinji Sakaguchi, Fumitake Saito, Ayumi Nakao, Hideki Yuki, Keiko Mitamura

**Affiliations:** 1 Department of Pulmonary Medicine, Eiju General Hospital, Tokyo, Japan; 2 Division of Pulmonary Medicine, Department of Medicine, Keio University School of Medicine, Tokyo, Japan; 3 Zama Children’s Clinic, Kanagawa, Japan; 4 Clinical Laboratory, Eiju General Hospital, Tokyo, Japan; 5 Department of Pediatrics, Eiju General Hospital, Tokyo, Japan; 6 Ichikawa Children’s Clinic, Kanagawa, Japan

## Abstract

*Mycoplasma pneumoniae* infection is conventionally diagnosed using serum antibody testing, microbial culture, and genetic testing. Recently, immunochromatography-based rapid mycoplasma antigen test kits have been developed and commercialised for rapid diagnosis of *M*. *pneumoniae* infection. However, as these kits do not provide sufficient sensitivity and specificity, a rapid test kit with improved accuracy is desired. The present prospective study evaluated a rapid *M*. *pneumoniae* diagnostic system utilizing a newly developed silver amplification immunochromatography (SAI) system. We performed dilution sensitivity test and the prospective clinical study evaluating the SAI system. The subjects of the clinical study included both children and adults. All patients suspected to have mycoplasma pneumonia (169 patients) were sequentially enrolled. Twelve patients did not agree to participate and 157 patients were enrolled in the study. The results demonstrate excellent performance of this system with 90.4% sensitivity and 100.0% specificity compared with real-time polymerase chain reaction. When compared with loop-mediated isothermal amplification (LAMP) methods, the results also demonstrate a high performance of this system with 93.0% sensitivity and 100.0% specificity. The SAI system uses a dedicated device for automatic analysis and reading, making it highly objective, and requires less human power, supporting its usefulness in clinical settings.

## Introduction

*Mycoplasma pneumoniae* is one of the most important causes of community-acquired pneumonia (CAP) in both children and adults^1,2^. As a causative pathogen, *M*. *pneumoniae* is detected as frequently as *Streptococcus pneumoniae* and *Haemophilus influenzae*^3^. While bacteriological culture is the gold standard for diagnosis, it is not suitable for rapid diagnosis, and oral bacteria sometimes contaminate the medium^4^. Furthermore, bacteriological cultures, especially of *M*. *pneumoniae* cannot be commonly performed in clinical settings due to technical difficulties.

Serum antibody tests, often used for laboratory examination, also have certain disadvantages^5^. Firstly, there is a time delay between the onset of infection and seroconversion of both *M*. *pneumoniae*-specific IgG and IgM. Secondly, serum tests continue to yield positive results for a long time, even after recovery. Therefore, it is often difficult to make a diagnosis solely based on the antibody titre of a single serum sample during the acute phase^6^. Moreover, it lacks diagnostic speed since definitive detection of *M*. *pneumoniae* generally requires titre elevation based on paired serum samples at a certain interval. Since *M*. *pneumoniae* is seasonally endemic and late diagnosis increases the risk of infection in the surroundings, an improved diagnostic system, which is rapid and practical, is warranted^5^.

To improve the diagnostic speed, new techniques for rapid diagnosis, such as loop-mediated isothermal amplification (LAMP) and immunochromatography-based rapid mycoplasma antigen test, have recently been proposed^7–10^. LAMP is a technique that rapidly amplifies DNA under isothermal conditions with high sensitivity^11^. However, LAMP is not used by primary care physicians thus far, partly due to its cost. Conventional immunochromatography-based rapid mycoplasma antigen test kits are problematic due to their low sensitivity^9^.

Recently, photographic development was applied to increase the sensitivity of immunochromatographic assays^12^. In this system, silver halide crystals act as a catalyst when sufficiently exposed to light and silver ions in the developer solution are reduced to metallic silver atoms. Our previous report evaluated highly sensitive silver amplification immunochromatography (SAI) systems for the rapid diagnosis of influenza^13^, which demonstrated superior sensitivity and specificity. In this prospective study, we report the clinical evaluation of a rapid mycoplasma diagnostic system using this new photographic development technology.

## Results

### Dilution sensitivity test

The dilution sensitivity test results are presented in Table 1. In terms of assessing the minimum dilution level for positivity, the control reagent testing was negative at 15 minutes and positive at 30 minutes with a 4-fold dilution of strain ATCC M129. The detection limit was 1.00 × 10^8^ copies/mL according to the mycoplasma DNA concentration. SAI system testing was positive with a 256-fold dilution; the limit of detection was 1.56 × 10^6^ copies/mL according to the mycoplasma DNA concentration. Therefore, the SAI system was 64-fold more sensitive than the control reagent. The SAI system was 16-fold more sensitive for strain ATCC FH and the clinical swab specimen, respectively, compared to the control reagent. Compared with the control reagent, the SAI system was capable of detecting all tested samples at lower antigen concentrations.

**Table 1:** Dilution sensitivity test.

Samples	Dilution rate	Copy numbers of Mycoplasma DNA (copies/mL)	SAI system	Control reagent	
			Result	Result at 15 minutes	Result at 30 minutes
ATCC M129	x2	2.00 × 10^8^	n. t.	+	+
	x4	1.00 × 10^8^	n. t.	−	+
	x8	5.00 × 10^7^	n. t.	−	−
	x16	2.50 × 10^7^	n. t.	−	−
	x32	1.25 × 10^7^	+	−	−
	x64	6.25 × 10^6^	+	n. t.	n. t.
	x128	3.13 × 10^6^	+	n. t.	n. t.
	x256	1.56 × 10^6^	+	n. t.	n. t.
	x512	7.81 × 10^5^	−	n. t.	n. t.
ATCC FH	x2	1.03 × 10^8^	n. t.	+	+
	x4	5.13 × 10^7^	n. t.	+	+
	x8	2.56 × 10^7^	n. t.	+	+
	x16	1.28 × 10^7^	n. t.	+	+
	x32	6.41 × 10^6^	n. t.	−	−
	x64	3.20 × 10^6^	n. t.	n. t.	n. t.
	x128	1.60 × 10^6^	+	n. t.	n. t.
	x256	8.01 × 10^5^	+	n. t.	n. t.
	x512	4.00 × 10^5^	−	n. t.	n. t.
Clinical specimen	x1	5.25 × 10^6^	+	+	+
	x2	2.63 × 10^6^	+	−	−
	x4	1.31 × 10^6^	+	−	−
	x8	6.57 × 10^5^	+	n. t.	n. t.
	x16	3.28 × 10^5^	+	n. t.	n. t.
	x32	1.64 × 10^5^	−	n. t.	n. t.
	x64	8.21 × 10^4^	−	n. t.	n. t.

n.t.: not tested, SAI: silver amplification immunochromatography.

### Cross-reactivity testing

No cross-reactivity was identified with other pathogens in this SAI system (Supplementary Table 1).

### Clinical study

**Figure 1:**
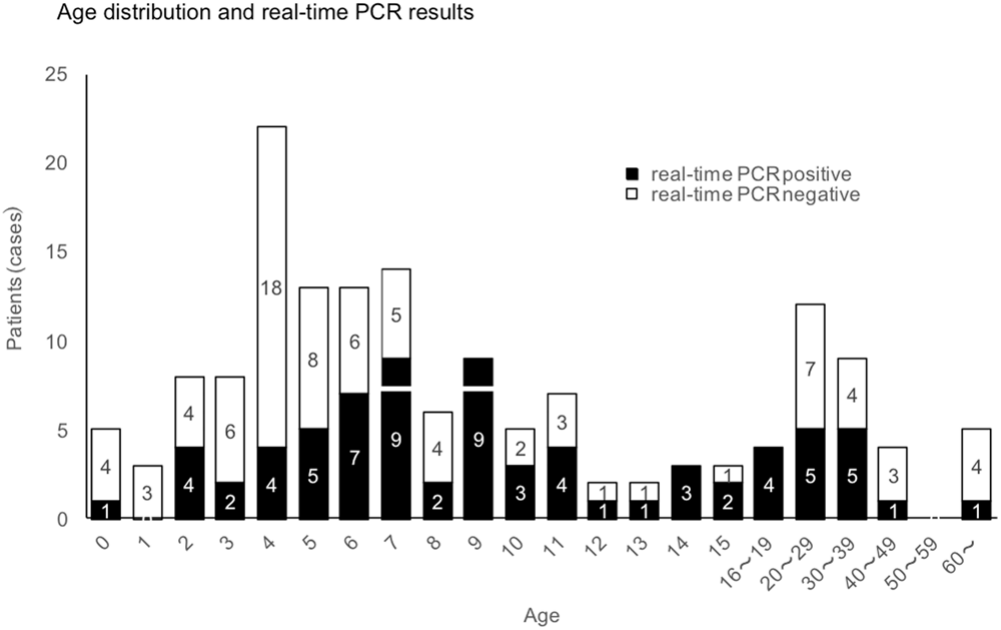
Age distribution and real-time PCR results. Age distribution and the performance of real-time polymerase chain reaction (PCR) are summarised in this figure. Both children and adults were enrolled in this study.

A total of 157 patients were enrolled. The age distribution and performance of real-time polymerase chain reaction (PCR) are summarised in Fig. 1. Table 2 summarises the clinical symptoms of patients participating in this study, stratified by age: 15 years and younger (123 children) vs. 16 years and older (34 adults; range, 16–93 years). Since the present study was designed with an inclusion criterion that required eligible patients to have a fever or cough, most of the enrolled patients (88.9%) had cough symptoms. In the 15 years and younger age group, the proportions of patients with a fever (38 °C or over), nasal discharge, sore throat, headache and general fatigue were 94.7%, 17.5%, 7.0%, 7.0%, and 1.8%, respectively, among 57 patients who tested positive and 89.4%, 39.4%, 4.5%, 0.0% and 3.0%, respectively, among 66 patients who were tested negative on real-time PCR analysis. In the 16 years and older age group, the proportions of patients with a fever (38 °C or over), nasal discharge, sore throat, headache and general fatigue were 100.0%, 50.0%, 56.3%, 18.8%, and 75.0%, respectively, among 16 patients who tested positive and 88.9%, 50.0%, 66.7%, 16.7%, and 55.6%, respectively, among 18 patients who tested negative on real-time PCR analysis. Among 123 eligible patients aged 15 years or younger, 117 patients were radiographically diagnosed with pneumonia. All 34 eligible patients aged 16 years or older were radiographically diagnosed with pneumonia. Table 3 summarises the sensitivity and specificity of the SAI system, control reagent, and the LAMP test, compared to real-time PCR as a reference. Of all 157 patients, 73 tested positive and 84 tested negative in the real-time PCR test. Among the 73 patients with positive real-time PCR test results, 47 tested positive for mycoplasma antigen and 26 were deemed to have undetectable results with the control reagent, which corresponded to a sensitivity of 64.4%. In contrast, 66 tested positive for the antigen and 7 were deemed to have undetectable results with the SAI system, which indicated a significantly higher sensitivity of 90.4% than the control reagent (P = 0.0002). Among the 84 patients with negative real-time PCR results, 76 tested negative with the control reagent, demonstrating a specificity of 90.5%, whereas all 84 tested negative with the SAI system. Thus, the SAI system exhibited a significantly higher specificity of 100.0% than the control reagent (P = 0.0078). Also, for lower respiratory tract infection, this SAI system showed high sensitivity and specificity. When compared with LAMP methods, the results also demonstrated a high performance of this system with 93.0% sensitivity and 100.0% specificity (Table 4).

**Table 2:** Clinical symptoms and lower respiratory tract infection.

Results		15 years and younger (123 cases)				16 years and older (34 cases)			
		real-time PCR positive (57 cases)		real-time PCR negative (66 cases)		real-time PCR positive (16 cases)		real-time PCR negative (18 cases)	
Sex	Male	26		41		12		10	
	Female	31		25		4		8	
Symptoms		cases	%	cases	%	cases	%	cases	%
cough		57	100.0%	66	100.0%	16	100.0%	16	88.9%
fever (38 °C or over)		54	94.7%	59	89.4%	16	100.0%	16	88.9%
nasal discharge		10	17.5%	26	39.4%	8	50.0%	9	50.0%
sore throat		4	7.0%	3	4.5%	9	56.3%	12	66.7%
headache		4	7.0%	0	0.0%	3	18.8%	3	16.7%
general fatigue		1	1.8%	2	3.0%	12	75.0%	10	55.6%
Lower respiratory tract infection		55	96.5%	62	93.9%	16	100.0%	18	100.0%

**Table 3:** Sensitivity and specificity compared with real-time PCR.

		SAI system		control reagent		LAMP		Total
		positive	negative	positive	negative	positive	negative	
real-time PCR	positive	66	7	47	26	70	3	73
	negative	0	84	8	76	1	83	84
Total		66	91	55	102	71	86	157
Sensitivity		90.4%		64.4%		95.9%		
95%CI		81.2–96.1%		52.3–75.3%		88.5–99.1%		
Specificity		100.0%		90.5%		98.8%		
95%CI		95.7–100%		82.1–95.8%		93.5–99.97%		

SAI: silver amplification immunochromatography, LAMP: loop-mediated isothermal amplification, 95%CI: 95% confidence interval.

**Table 4:** Sensitivity and specificity compared with LAMP.

		SAI system		control reagent		Total
		positive	negative	positive	negative	
LAMP	positive	66	5	46	25	71
	negative	0	86	9	77	86
Total		66	91	55	102	157
Sensitivity		93.0%		64.8%		
95%CI		84.3–97.7%		52.5–75.8%		
Specificity		100%		89.5%		
95%CI		95.8–100%		81.1–95.1%		

SAI: silver amplification immunochromatography, LAMP: loop-mediated isothermal amplification, 95%CI: 95% confidence interval.

Among the 73 patients who tested positive with real-time PCR, the LAMP test yielded positive results for 70 patients and negative results for 3 patients. Among 84 patients who tested negative with real-time PCR, the LAMP test yielded positive results for one patient. The SAI system showed equivalent sensitivity and specificity to the LAMP test (sensitivity P = 0.125, specificity P = 0.5).

**Figure 2:**
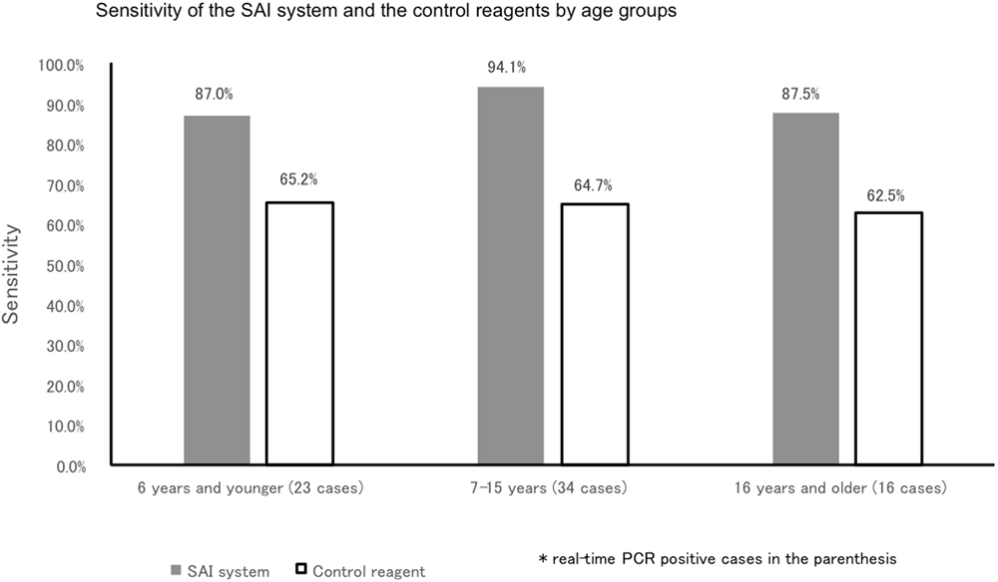
Sensitivity of the SAI system and the control reagents by age groups. The sensitivity of the SAI system, when compared with the control reagent by age stratum, was high and stable, regardless of the age of the patient. SAI: silver amplification immunochromatography.

We also evaluated the copy numbers of mycoplasma DNA in the SAI system. The SAI system demonstrated marked discrimination ability in terms of the copy numbers (Supplementary Figure 1). The sensitivity of the SAI system, when compared with the control reagent by age stratum, was high and stable, regardless of the age of the patient (Fig. 2).

**Figure 3:**
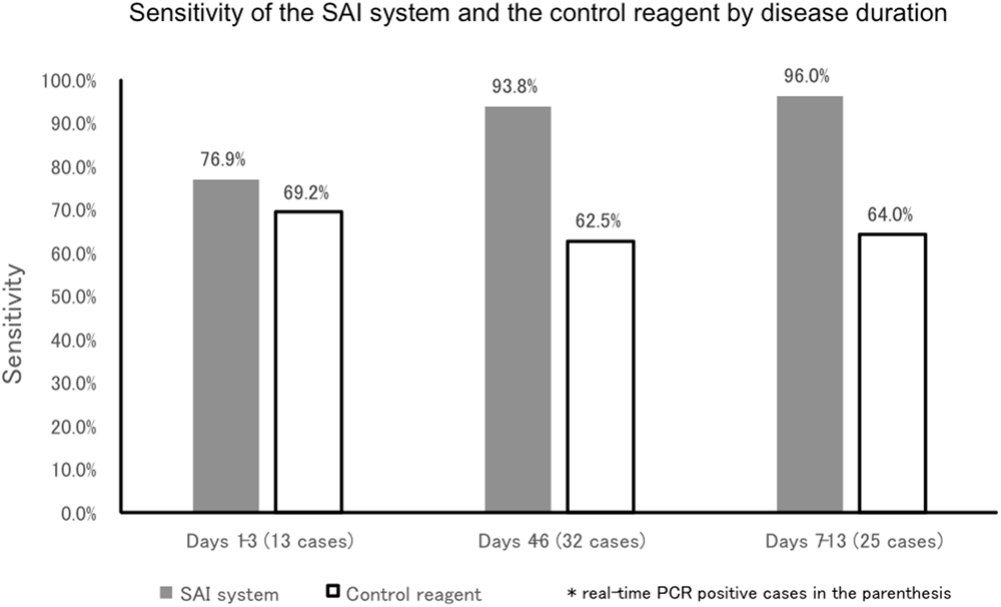
Sensitivity of the SAI system and the control reagent by disease duration. The sensitivity of SAI system according to the disease duration was favourable, with 76.9% during days 1–3, 93.8% for days 4–6, and 96.0% for day 7 onwards, compared with 69.2%, 62.5%, and 64.0%, respectively, as detected by the control reagent. SAI: silver amplification immunochromatography.

We also evaluated disease duration as the number of days from the onset of symptoms. The sensitivity of the SAI system according to the disease duration was also favourable, with 76.9% during days 1–3, 93.8% for days 4–6, and 96.0% for day 7 onwards, compared with 69.2%, 62.5%, and 64.0%, respectively, as detected by the control reagent (Fig. 3).

## Discussion

To the best of our knowledge, this is the first study to evaluate the efficacy of the SAI system as a rapid diagnostic tool for *M*. *pneumoniae* infection. *M*. *pneumoniae* is a clinically important pathogen among children and adults that is periodically endemic^2,5^. In order to prevent further spread to the community, a rapid and simple diagnostic tool is required. Detection of *M. pneumoniae* by culture isolation or by paired serum samples requires time, and culture isolation is not feasible in general medical institutions^4^. Gene amplification methods, such as PCR testing and the LAMP method require expensive machinery, and are not practical for primary care physicians, who diagnose *M. pneumoniae* infection most frequently. Thus, conventional immunochromatography-based rapid mycoplasma antigen test kits were developed for rapid and facile methods^9,10^. Although these kits do not require specific machinery, the sensitivity and specificity has been demonstrated to be low, and further improvements to ensure precise and accurate diagnosis are warranted^10^. To overcome these disadvantages, the SAI system was developed for the detection of *M*. *pneumoniae* (Supplementary Figure 2). SAI improved the detection ability by applying photographic development technology to immunochromatography, as we have previously reported in a diagnostic study of influenza^13^.

There are several advantages that demonstrate the usefulness of the SAI system. Firstly, this SAI system is easy to use in clinical practice and its speed is noteworthy. It takes 15 minutes to judge positivity without costly machinery^12^. Its user-friendliness will decrease the burden of diagnosis in clinical settings.

Secondly, in the clinical study, the SAI system exhibited sufficient sensitivity and specificity, which was higher than conventional immunochromatography, and equivalent to the LAMP test. The preceding study highlighted the low sensitivity of conventional immunochromatographic test; however, this SAI system has solved this issue^9^. Regarding specificity, the control reagent produced false positives in 8/55 patients (14.5%) with negative PCR results, whereas the SAI system did not produce any false positive results. This specificity finding also supports the usefulness of the SAI system as an aid for the accurate diagnosis of mycoplasma infection. The SAI system also showed higher sensitivity in the dilution sensitivity test. The dilution sensitivity test demonstrated that the SAI system has a sensitivity that is at least 16-fold higher than the control reagent for both cultured strains and throat swab specimens. Since *M*. *pneumoniae* predominantly infects and proliferates in the lower respiratory tract, the bacterial load is lower in the upper airway, which is the site of sampling^14^. Therefore, the improved sensitivity of the SAI system will contribute to an improved detection rate with clinical specimens. Although the detection limit of SAI system is higher in comparison with other immunochromatographic methods^10^, we suppose that direct comparison of the detection limits should be performed under the same conditions.

Thirdly, this research targeted both children and adults. Regardless of age groups, the SAI system proves sufficient diagnostic ability (Fig. 2). Moreover, the sensitivity of the SAI system was higher than the control reagent regardless of disease duration. This study is unique in evaluating both children and adults. Since *Mycoplasma pneumoniae* can infect both children and adults, our results reflect the actual clinical setting.

Regarding clinical symptoms, a previous study reported that patients with suspected respiratory mycoplasma infection had a high prevalence of cough, fever, nasal symptom, headache, and pharyngeal symptoms^5^. The present clinical evaluation selected a subject group of patients with fever or cough and suspected *M. pneumoniae* infection, which eventually consisted mostly of those with both fever and cough, regardless of the real-time PCR test result. As mycoplasma infection is less associated with clear nasal discharge or purulent sputum, the present evaluation supported a lower tendency of nasal discharge, which was seen in 17.5% and 39.4% of patients aged 15 years or younger with positive and negative real-time PCR results, respectively.

There are limitations to this study. This study was conducted only at three institutions. In particular, the study for adults was conducted at a single centre. In the future, further evaluation of this diagnostic tool will be required with a larger sample size from multiple centres.

In conclusion, the SAI system is highly objective as it automatically produces results in a fixed time window of 15 minutes, and provides higher sensitivity and specificity than the conventional immunochromatographic test. We believe that the SAI system is highly useful as point-of-care testing for *M. pneumoniae* infection in various clinical settings, including clinics and hospitals in communities.

## Methods

### Test reagent and control reagent systems

The SAI system consists of a reagent Quick Chaser® Auto Myco (Mizuho Medy, Saga, Japan) or FUJI DRI-CHEM IMMUNO AG Cartridge Myco (Fujifilm, Kanagawa, Japan) combined with a dedicated analyser Quick Chaser Immuno Reader (Mizuho Medy, Saga, Japan) or FUJI DRI-CHEM IMMUNO AG1 (Fujifilm, Kanagawa, Japan). The reagent system, which utilised the rapid mycoplasma antigen test kit Ribotest® Mycoplasma (Asahi Kasei Pharma), was used as a control.

### Samples for dilution sensitivity test

The dilution sensitivity test was performed using three samples, including strains ATCC M129 and ATCC FH, and a throat swab specimen from a patient who tested positive for mycoplasma according to the control reagent.

### Patient enrolment

This study enrolled patients, aged 0 or over, who visited either Eiju General Hospital (Department of Pediatrics or Pulmonary Medicine) or Zama Children’s Clinic between December 25, 2015 and December 31, 2016. Patients presenting with fever or cough, who were clinically suspected to have *M. pneumoniae* infection and signed informed consent documents for participation in this study were ultimately enrolled. This study was conducted upon approval by the Ethical Committee of Eiju General Hospital, an affiliate of Research Institute for Life Extension (2015-8) (UMIN000025746), and carried out in accordance with the guidelines approved by that committee. Informed consent was obtained from all subjects prior to the study. During the study period, all patients suspected to have mycoplasma pneumonia (169 patients) were sequentially enrolled. Twelve subjects (11 children and 1 adult) did not agree to participate in the study. Finally, a total of 157 patients were enrolled.

### Procedures of the SAI system

The SAI system was performed according to the procedure described as follows:

- Take a mucosal epidermis sample by rubbing the posterior pharyngeal wall several times with a sterile swab.
- Elute the specimen adhering to the swab into the extraction reagent solution in the extraction tube.
- Drop the sample onto the instilling section of the test cartridge.
- Set the cartridge to the instrument and start measurement.
- The result will be displayed automatically after 15 minutes.

### Procedures of the control reagent system

The Ribotest® kit was used, according to the manufacturer’s instruction. Since the instructions recommend a judgment be made between 15 and 30 minutes after initiation, the result was visually determined and recorded at both 15 and 30 minutes.

### Dilution sensitivity test

Each of the strains and the throat swab specimen were suspended in saline (stock suspension) and a series of 2-fold serial dilutions were subsequently prepared. Using the dilution series, the assay was performed with swab sampling according to the procedure outlined for the SAI system and the control reagent, respectively. We compared the detection limits between the two reagent systems. In addition, the mycoplasma DNA copy numbers in each stock suspension were determined by real-time PCR and the copy numbers in each dilution were calculated. DNA was extracted using QIAamp^®^ DNA Mini Kit (QIAGEN). Once SYBR^®^ Green I (SYBR^®^ Premix Dimer Eraser™: TaKaRa) and primers were added, real-time PCR was performed using a Thermal Cycler Dice^®^ Real Time System TP800 (TaKaRa). We made plasmid DNA of *M. pneumoniae* ATCC29342 in the region of 16S rRNA (GeneBank accession AF132741 position 1202–1391). We used this as PCR standard. In PCR, we used the same primer as Kessler *et al*. reported^15^. We evaluated copy numbers in the SAI system using the residual liquids of the same specimen. We performed three independent experiments in the dilution sensitivity test.

### Clinical study

From each patient, throat swab specimens were collected three times, with ancillary swabs specific for each reagent. We rubbed the three swabs and mixed properly after specimen collection to average the copy numbers of *M. pneumoniae* among the swabs. These specimens were evaluated by the SAI system, the control reagent, and LAMP test, respectively. Using the real-time PCR test as a reference, the sensitivity and specificity were calculated for the SAI system, the control reagent, and the LAMP test. Sensitivity and specificity were statistically compared between the SAI system, the control reagent, and the LAMP test reagent using McNemar’s test. At the time of diagnosis, a physician reviewed chest X-ray findings and diagnosed the lower respiratory tract infection. The diagnoses were retrospectively confirmed by two independent researchers; in cases of discrepancies, the diagnoses were determined by consensus.

### Data Availability

The datasets generated during and/or analysed during the current study are available from the corresponding author on reasonable request.

## Electronic supplementary material

Supplementary Table

Supplementary Figure

Blast search
