# Supplementary material for: Clinical Evaluation of the Immunochromatographic System Using Silver Amplification for the Rapid Detection of Mycoplasma pneumoniae
Source: Sci Rep. 2018 Jan 23;8:1430. doi: 10.1038/s41598-018-19734-y (PMC5780467; doi:10.1038/s41598-018-19734-y)
Supplement: Supplementary file 1 — Supplementary Table [file 41598_2018_19734_MOESM1_ESM.doc]

**Title**: Clinical evaluation of the immunochromatographic system using silver amplification for the rapid detection of *Mycoplasma pneumoniae*

**Author**

Ho Namkoong1)2), Masahiko Yamazaki 3), Masami Ishizaki 3), Ikumi Endo 4), Noriaki Harada 4),Megumi Aramaki5), Yuko Tanaka5), Sachiko Kaburagi5), Masataka Ichikawa 6) , Takanori Ohata1), Shinji Sakaguchi1), Fumitake Saito1), Ayumi Nakao 5), Hideki Yuki1), Keiko Mitamura 5)

1） Department of Pulmonary Medicine, Eiju General Hospital

2） Division of Pulmonary Medicine, Department of Medicine, Keio University School of Medicine

3) Zama Children’s Clinic

4) Clinical Laboratory, Eiju Genaral Hospital

5) Department of Pediatrics, Eiju General Hospital

6) Ichikawa Children’s Clinic

Correspondence

Ho Namkoong, M.D.

Division of Pulmonary Medicine, Department of Medicine, Keio University School of Medicine

35 Shinanomachi, Shinjuku-ku, Tokyo 160-8582, Japan

Tel: +81-3-3353-1211 (ext. 62310), Fax: +81-3-3353-2502

E-mail: hounamugun@gmail.com

Supplementary Table No cross-reactivity with other pathogens

| **Other Mycoplasma** |
| --- |
| *Mycoplasma fermentans, Mycoplasma genitalium, Mycoplasma orale, Mycoplasma salivarium, Mycoplasma buccale, Mycoplasma hominis, Mycoplasma faucium, Mycoplasma penetrans, Mycoplasma laidlawii, Ureaplasma urealyticum* |
| **Bacteria** |
| *Candida albicans, Citrobacter freundii, Escherichia coli, Haemophilus influenzae, Klebsiella pneumoniae, Listeria monocytogenes, Legionella pneumophila, Moraxella catarrhalis, Proteus mirabilis, Pseudomonas aeruginosa, Serratia marcescens, Staphylococcus aureus, Staphylococcus epidermidis, Streptococcus anginosus, Streptococcus mutans, Streptococcus pneumoniae, Streptococcus pyogenes* (group A)*, Streptococcus agalactiae* (group B)*, Streptococcus* (group C) |
| **Viruses** |
| Influenzavirus A, Influenzavirus B, Adenovirus type 1, Adenovirus type 2, Adenovirus type 3, Adenovirus type 4, Adenovirus type 5, Adenovirus type 7, Human Coronavirus, Coxsackie virus A9, Coxsackie virus B5, Human Echovirus 9, Herpes simplexvirus type 1, Human metapneumovirus, Mumps virus, Human parainfluenza virus 1, Rhinovirus 8, Respiratory syncytial virus A, Respiratory syncytial virus B |
