# Supplementary material for: Clinical Evaluation of the Immunochromatographic System Using Silver Amplification for the Rapid Detection of Mycoplasma pneumoniae
Source: Sci Rep. 2018 Jan 23;8:1430. doi: 10.1038/s41598-018-19734-y (PMC5780467; doi:10.1038/s41598-018-19734-y)
Supplement: Supplementary file 2 — Supplementary Figure [file 41598_2018_19734_MOESM2_ESM.doc]

**Title**: Clinical evaluation of the immunochromatographic system using silver amplification for the rapid detection of *Mycoplasma pneumoniae*

**Author**

Ho Namkoong1)2), Masahiko Yamazaki 3), Masami Ishizaki 3), Ikumi Endo 4), Noriaki Harada 4),Megumi Aramaki5), Yuko Tanaka5), Sachiko Kaburagi5), Masataka Ichikawa 6) , Takanori Ohata1), Shinji Sakaguchi1), Fumitake Saito1), Ayumi Nakao 5), Hideki Yuki1), Keiko Mitamura 5)

1） Department of Pulmonary Medicine, Eiju General Hospital

2） Division of Pulmonary Medicine, Department of Medicine, Keio University School of Medicine

3) Zama Children’s Clinic

4) Clinical Laboratory, Eiju Genaral Hospital

5) Department of Pediatrics, Eiju General Hospital

6) Ichikawa Children’s Clinic

Correspondence

Ho Namkoong, M.D.

Division of Pulmonary Medicine, Department of Medicine, Keio University School of Medicine

35 Shinanomachi, Shinjuku-ku, Tokyo 160-8582, Japan

Tel: +81-3-3353-1211 (ext. 62310), Fax: +81-3-3353-2502

E-mail: hounamugun@gmail.com

**Supplementary Figure 1 Copy numbers of mycoplasma DNA in the SAI system**

The SAI system demonstrated discrimination ability in terms of the copy numbers.

SAI: silver amplification immunochromatography

**Supplementary Figure 2 Principle of silver amplification immunochromatography**
